# Bilirubin reduces visceral obesity and insulin resistance by suppression of inflammatory cytokines

**DOI:** 10.1371/journal.pone.0223302

**Published:** 2019-10-02

**Authors:** Ryoko Takei, Tomoaki Inoue, Noriyuki Sonoda, Motoyuki Kohjima, Misato Okamoto, Ryuichi Sakamoto, Toyoshi Inoguchi, Yoshihiro Ogawa

**Affiliations:** 1 Department of Medicine and Bioregulatory Science, Graduate School of Medical Sciences, Kyushu University, Fukuoka, Japan; 2 Fukuoka City Health Promotion Support Center, Fukuoka, Japan; 3 Department of Molecular and Cellular Metabolism, Graduate School of Medical and Dental Sciences, Tokyo Medical and Dental University, Tokyo, Japan; Tohoku University, JAPAN

## Abstract

**Objective:**

Although previous studies have reported a negative relationship between serum bilirubin concentration and the development of diabetes mellitus (DM), the relationship between bilirubin and insulin resistance has not been thoroughly assessed. This study was designed to determine the relationships between bilirubin, body fat distribution, and adipose tissue inflammation in patients with type 2 DM and the effect of bilirubin in an obese animal model.

**Method:**

Body fat distribution was measured using an abdominal dual bioelectrical impedance analyzer in patients with type 2 DM. We also measured glycemic control, lipid profile, serum bilirubin concentration and other clinical characteristics, and determined their relationships with body fat distribution. In the animal study, biliverdin (20 mg/kg daily) was orally administered to high-fat diet (HFD)-induced obese (DIO) mice for 2 weeks, after which intraperitoneal insulin tolerance testing was performed. Then, adipocyte area, adipocytokine expression, and macrophage polarization were evaluated in epididymal adipose tissues.

**Results:**

In the clinical study, univariate analysis showed that a lower bilirubin concentration was significantly correlated with higher body mass index, waist circumference, triglyceride, uric acid, creatinine, visceral fat area and lower HDL-C. In multivariate analyses, bilirubin concentration significantly correlated with diastolic blood pressure, creatinine, and visceral fat area. However, there was no association between bilirubin concentration and subcutaneous fat area. In the animal study, DIO mice treated with biliverdin had smaller adipocytes than untreated DIO mice and biliverdin improved HFD-induced insulin resistance. Biliverdin treatment reversed the higher gene expression of *Cd11c*, encoding an M1 macrophage marker, and *Tnfa*, encoding the proinflammatory cytokine tumor necrosis factor-α, in the adipose tissues of DIO mice. These data suggest biliverdin administration alleviates insulin resistance by ameliorating inflammation and the dysregulation of adipocytokine expression in adipose tissues of DIO mice.

**Conclusions:**

Bilirubin may protect against insulin resistance by ameliorating visceral obesity and adipose tissue inflammation.

## Introduction

Obesity is a risk factor for type 2 diabetes mellitus [1, 2], and recent studies have shown that body fat distribution may be more important than overall adiposity. In particular, visceral fat is a strong and independent predictor of metabolic dysfunction [3, 4], and was reported to be involved the pathogenesis of type 2 diabetes mellitus [5]. Excessive production of proinflammatory cytokines by visceral adipose tissue macrophages is considered critical for the obesity-associated adipose tissue inflammation [6, 7] that leads to insulin resistance. We previously reported that the prevalence of diabetic complications was markedly lower in diabetic patients with Gilbert syndrome, characterized by congenital hyperbilirubinemia [8]. In addition, we showed that the serum bilirubin concentration was inversely associated with hemoglobin A1c (HbA1c) and the prevalence of type 2 diabetes mellitus in a large cross-sectional study [9]. Hinds et al. [10] reported that humanized Gilbert’s syndrome mice had increased PPARα activation and reduced hepatic fat accumulation induced by a high fat diet (HFD). While biliverdin reductase-A (BVRA) protected against hepatic steatosis via PPARα activation [11], BVRA knockout mice on a HFD were glucose insensitive [11] and BVRA was lower in obese humans with insulin resistance [12]. Although previous studies suggested that bilirubin functions as an antioxidant [13] and an important modulator of chronic inflammation in metabolic syndrome and diabetes [14], the underlying mechanism of the relationships between bilirubin and insulin resistance, body fat distribution, and adipose tissue inflammation in diabetes mellitus have not been fully characterized. Therefore, in this study we elucidated these relationships in patients with type 2 diabetes mellitus and in an animal model.

## Materials and methods

### Clinical study

We enrolled 176 Japanese patients (90 men and 86 women) with type 2 diabetes mellitus who were admitted to the metabolic ward of Kyushu University Hospital between June 2017 and December 2018. A diagnosis of diabetes mellitus was confirmed using the criteria of the American Diabetes Association/World Health Organization (ADA/WHO), or by confirming a medical history of diabetes. The clinical study was performed in accordance with the Declaration of Helsinki and approved by the Clinical Ethics Committee of Kyushu University Hospital (No. 29–33). Written informed consent was obtained from each patient. Patients undergoing therapy for chronic hepatitis, liver cirrhosis, or liver cancer, who had a prior history of liver cancer, who had serum aspartate aminotransferase (AST) or alanine aminotransferase (ALT) concentrations greater than three times the upper limit of the normal range (>120 U/L), or who had a serum total bilirubin concentration >3.0 mg/dL, were excluded.

All patients underwent clinical evaluation and laboratory assessment. Body mass index (BMI) was calculated as body mass (kg) divided by height squared (m^2^). Waist circumference (WC) was measured at the midpoint between the upper end of the iliac crest and the lower end of the 12th rib, at the end of each subject’s normal expiration, using an anthropometric tape. The visceral fat area (VFA) and subcutaneous fat area (SFA) were measured using an abdominal dual bioelectrical impedance analyzer (Dualscan HDS-2000; Omron Healthcare Co., Kyoto, Japan). The fasting concentrations of plasma glucose, HbA1c (National Glycohemoglobin Standardization Program), total cholesterol (TC), high-density lipoprotein-cholesterol (HDL-C), triglycerides (TG), uric acid (UA), bilirubin, and creatinine were measured. HbA1c levels (%) were converted to International Federation of Clinical Chemistry and Laboratory Medicine mmol/mol units using the NGSP converter for HbA1c (http://www.ngsp.org/convert1.asp).

### Animal study

#### Animals and experimental protocol

Five-week-old male C57Bl/6J mice were purchased from Clea Japan Inc. (Tokyo, Japan). Mice were housed in colony cages under a 12-h light/12-h dark cycle, with free access to tap water and chow (Clea Japan Inc.). At 8 weeks of age, they started consuming either a control diet (CD; 74% carbohydrate, 14% protein, and 12% fat) or a HFD (20% carbohydrate, 18% protein, and 62% fat) for 8 weeks. At 16 weeks of age, half of the mice fed the HFD (n = 16) or CD (n = 16) were randomly chosen to be switched to a powdered diet (Clea Japan Inc.) supplemented with biliverdin (20 mg/kg) (Frontier Scientific, Logan, UT, USA) for 2 weeks, while the remaining mice consumed a control powdered diet that did not contain biliverdin, for the same time period (S1 Fig), as described previously [15]. All protocols were reviewed and approved by the Committee on the Ethics of Animal Experiments, Graduate School of Medical Science, Kyushu University.

#### Measurement of blood glucose and intraperitoneal insulin tolerance testing

Blood samples were obtained from the tail vein of each mouse. Plasma glucose and insulin concentrations were determined using the glucose oxidase method and an enzyme-linked immunosorbent assay (ELISA; Morinaga Institute of Biological Science, Yokohama, Japan), respectively. Plasma adiponectin and leptin concentrations were also determined by ELISA (Wako, Osaka, Japan). The degree of insulin resistance was assessed using an insulin tolerance test (ITT). Briefly, mice were injected with 2 U/kg human biosynthetic insulin (Novo Nordisk, NJ, USA), then blood samples were collected at 0, 15, 30, 60, 90, and 120 min, and their glucose concentrations were measured, as described above. Homeostasis model assessment of insulin resistance (HOMA-IR) was determined as described previously [16, 17].

#### Histologic analysis of white adipose tissue (WAT)

WAT was collected from the intra-abdominal perigonadal fat pad, which was previously shown to be metabolically significant and a site at which inflammation develops during obesity, and weighed [18]. Bilateral perigonadal fat pads were dissected and weighed, and the fat pad mass was calculated as a percentage of body mass. To estimate adipocyte size [19], formalin-fixed, paraffin-embedded WAT sections were stained with hematoxylin and eosin, and 100 adipocytes per mouse were quantitatively evaluated by microscopy.

#### RNA extraction and quantitative RT-PCR

Total RNA was extracted from frozen epididymal adipose samples using a RNeasy Adipose Tissue Mini Kit (Qiagen, Chatsworth, CA, USA), according to the manufacturer’s instructions. The extracted RNA (1 μg) was reverse transcribed to single-stranded cDNA using a QuantiTect Reverse Transcription Kit (Qiagen, Valencia, CA, USA). Specific mRNA expression levels were measured by quantitative RT-PCR using iTaq SYBR Green mix (Bio-Rad) and a Bio-Rad Chromo 4/Opticon cycler. PCR reactions for each target cDNA were performed using the conditions shown in S1–S3 Tables. The linearity of the amplifications as a function of cycle number was assessed in preliminary experiments. The mRNA expression of each gene was normalized to the expression of the reference gene *β-actin*.

### Statistical analysis

All statistical analyses were performed using JMP statistical software, Version 13 (SAS Institute Inc., Cary, NC, USA). For the clinical study, continuous variables were analyzed using Spearman’s rank correlation and categorical variables using the Mann–Whitney *U*-test for univariate analysis of the relationship between serum bilirubin concentration, body fat distribution and each parameter. Multivariate linear regression analyses were conducted to control for potential confounders. Gender was coded as a dummy variable. Continuous data are summarized as medians and interquartile ranges (IQR) and categorical variables as absolute numbers (%). For the animal study, all data are expressed as means ± SEM. Statistical analysis was performed using the Student’s *t*-test or one-way analysis of variance (ANOVA), followed by Fisher’s protected least significant difference test. *P* < 0.05 was considered to represent statistical significance.

## Results

### Clinical study

The clinical, anthropometric, and metabolic characteristics of the clinical study cohort are shown in Table 1.

**Table 1:** Demographic and clinical characteristics of the patient cohort (N = 176).

Patient characteristics	
Age, years	60 (52–70)
Sex, male/female, %	90 (51.1)/86 (48.9)
Body mass index, kg/m^2^	25.5 (22.1–28.9)
WC, cm	93 (84–101)
SBP, mmHg	128 (112–140)
DBP, mmHg	76 (67–84)
Fasting plasma glucose, mg/dl	139 (117–177)
HbA1c, % (mmol/mol)	8.1 (7.2–9.5) (65 (55–80))
Total cholesterol, mg/dl	180 (154–210)
HDL-C, mg/dl	46 (37–56)
TG, mg/dl	130 (88–195)
UA, mg/dl	5.6 (4.6–6.5)
Bilirubin, mg/dl	0.8 (0.6–1.0)
Cre, mg/dl	0.71 (0.57–0.90)
VFA, cm^2^	85 (57–117)
SFA, cm^2^	200 (135–277)

Categorical variables are presented as a number (%) or median (lower quartile–upper quartile). WC; waist circumference; SBP, systolic blood pressure; DBP, diastolic blood pressure; HbA1c, hemoglobin A1c; HDL-C, high-density lipoprotein cholesterol; TG, triglycerides; UA, uric acid; Cre, creatinine; VFA, visceral fat area; SFA, subcutaneous fat area.

The relationships between serum bilirubin concentration and other variables are shown in Table 2. HDL-C was positively associated with serum bilirubin concentration, whereas BMI, WC, TG, UA, Cre, and VFA were inversely associated with serum bilirubin concentration. Even after adjustment for potential confounders, the serum bilirubin concentration was inversely associated with VFA (Table 2). The relationships between body fat distribution and other variables are shown in S4 Table. After adjustment, VFA was correlated with age, sex, WC, HbA1c, HDL-C, bilirubin and Cre. However, there was no association between serum bilirubin concentration and SFA (Table 2 and S4 Table).

**Table 2:** Correlations between serum bilirubin concentration and other variables.

	Univariate		Multivariate	
Variables	ρ	p value	β	p value
Age	0.052	0.493	0.128	0.159
Sex, female	-0.011	0.882	-0.163	0.061
Body mass index	-0.168	0.026	0.180	0.402
WC	-0.177	0.019	0.212	0.407
SBP	0.064	0.399	-0.085	0.401
DBP	0.101	0.183	0.205	0.048
Fasting plasma glucose	-0.036	0.632	-0.104	0.236
HbA1c	-0.010	0.897	0.045	0.616
Total cholesterol	-0.030	0.692	0.113	0.250
HDL-C	0.246	0.001	0.050	0.568
TG	-0.268	0.001	-0.168	0.091
UA	-0.200	0.008	-0.079	0.372
Cre	-0.160	0.034	-0.217	0.012
VFA	-0.191	0.011	-0.331	0.044
SFA	-0.130	0.085	-0.200	0.321

WC; waist circumference; SBP, systolic blood pressure; DBP, diastolic blood pressure; HbA1c, hemoglobin A1c; HDL-C, high-density lipoprotein cholesterol; TG, triglycerides; UA, uric acid; Cre, creatinine; VFA, visceral fat area; SFA, subcutaneous fat area.

### Animal study

#### Body mass, fasting glucose concentration, and epididymal fat mass

To explore the potential mechanisms underpinning the associations identified in the clinical study, we conducted an animal study to determine whether bilirubin reduced adipose tissue mass and improved obesity-induced insulin resistance. As shown in S1 Fig, there was a significant difference in body mass between CD-fed and HFD-fed mice between 8 and 16 weeks of age. At 16 weeks of age, blood glucose was significantly higher in HFD-fed than in CD-fed mice (Table 3). From this time point, biliverdin (20 mg/kg daily) was orally administered to half of the HFD-fed mice for 2 weeks. At 18 weeks of age after feeding with a control powdered diet supplemented with or without biliverdin for 2 weeks (S1 Fig), there was no difference in fasting and fed blood glucose between CD-fed mice, HFD-fed mice, and biliverdin-treated HFD-fed mice (Table 3). As shown in Table 3, the body mass of the HFD-fed mice was significantly higher than that in age-matched CD-fed mice at 18 weeks of age, but was not significantly affected by biliverdin treatment. The fat pad mass was higher in untreated HFD-fed mice than in age-matched CD-fed mice after 2 weeks of treatment (Table 3), but there was no difference in fat pad mass between CD-fed mice and biliverdin-treated HFD-fed mice.

**Table 3:** Effects of high-fat diet and biliverdin on body mass and metabolic indices in mice.

	Control	HFD	HFD + BVD
Baseline (16weeks)			
Body weight (g)	27.5 ± 0.3	37.6 ± 0.6*	37.4 ± 1.1*
Fed glucose (mg/dl)	155 ± 14	194 ± 10*	190 ± 9.3*
Two weeks after treatment(18weeks)			
Body weight (g)	29.2 ± 0.5	31.7 ± 0.5*	31.0 ± 0.7*
Fasting glucose (mg/dl)	94 ± 9.0	89 ± 3.5	93 ± 3.5
Fed glucose (mg/dl)	177 ± 6.7	172 ± 4.0	165 ± 11
Fasting insulin (ng/ml)	0.32 ± 0.14	0.58 ± 0.13	0.37 ± 0.09
HOMA-IR	1.7 ± 0.42	3.3 ± 0.73*	2.2 ± 0.47
Fat pad mass (%)	0.75 ± 0.22	1.34 ± 0.12*	1.18 ± 0.14

Control, control diet-fed mice; HFD, high-fat diet (HFD)-fed mice; HFD + BVD, HFD-fed mice treated with biliverdin. Data are means ± SEM (n = 8).

*P < 0.05 *vs* control (ANOVA).

#### Effect of biliverdin treatment on insulin resistance

**Fig 1:**
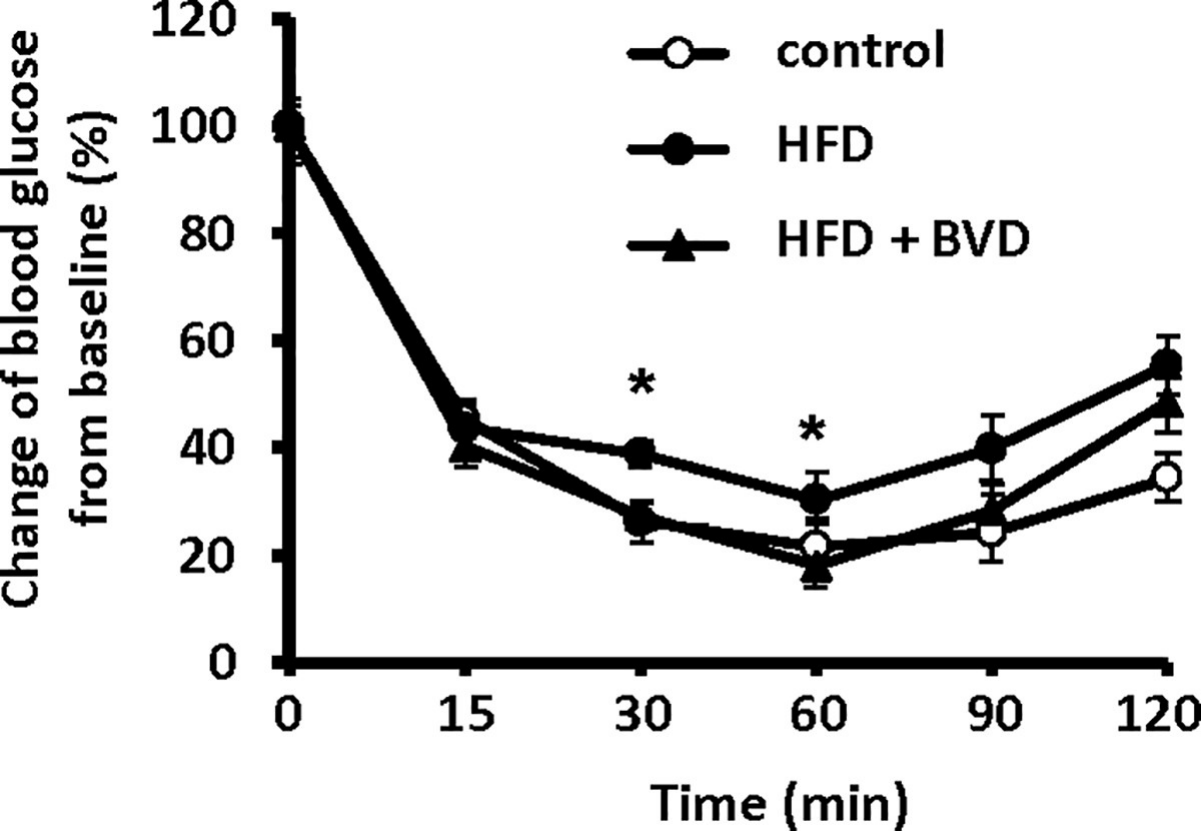
Effects of biliverdin (BVD) on HFD-induced insulin resistance. Insulin tolerance testing was performed in mice after 2 weeks of treatment. Blood glucose concentrations are expressed as percentages of basal blood glucose. Control mice (○), HFD mice (●), and HFD mice treated with biliverdin (BVD) (▲). Results are expressed as means ± SEM (n = 8). *P < 0.05 HFD mice *vs* HFD mice treated with BVD (ANOVA).

Next, we determined the effect of biliverdin treatment on insulin resistance. HOMA-IR was markedly increased in untreated HFD-fed mice compared with age-matched CD-fed mice after 2 weeks of treatment (Table 3), but there was no difference in HOMA-IR between CD-fed mice and biliverdin-treated HFD-fed mice. During ITTs, although the blood glucose concentration of untreated HFD-fed mice was higher than that of biliverdin-treated HFD-fed mice at the 30 and 60-min time points, there were no significant differences between CD-fed mice and biliverdin-treated HFD-fed mice (Fig 1). These data suggest that biliverdin treatment alleviates HFD-induced insulin resistance.

#### Effect of biliverdin treatment on adipose tissue inflammation and adipocyte size

**Fig 2:**
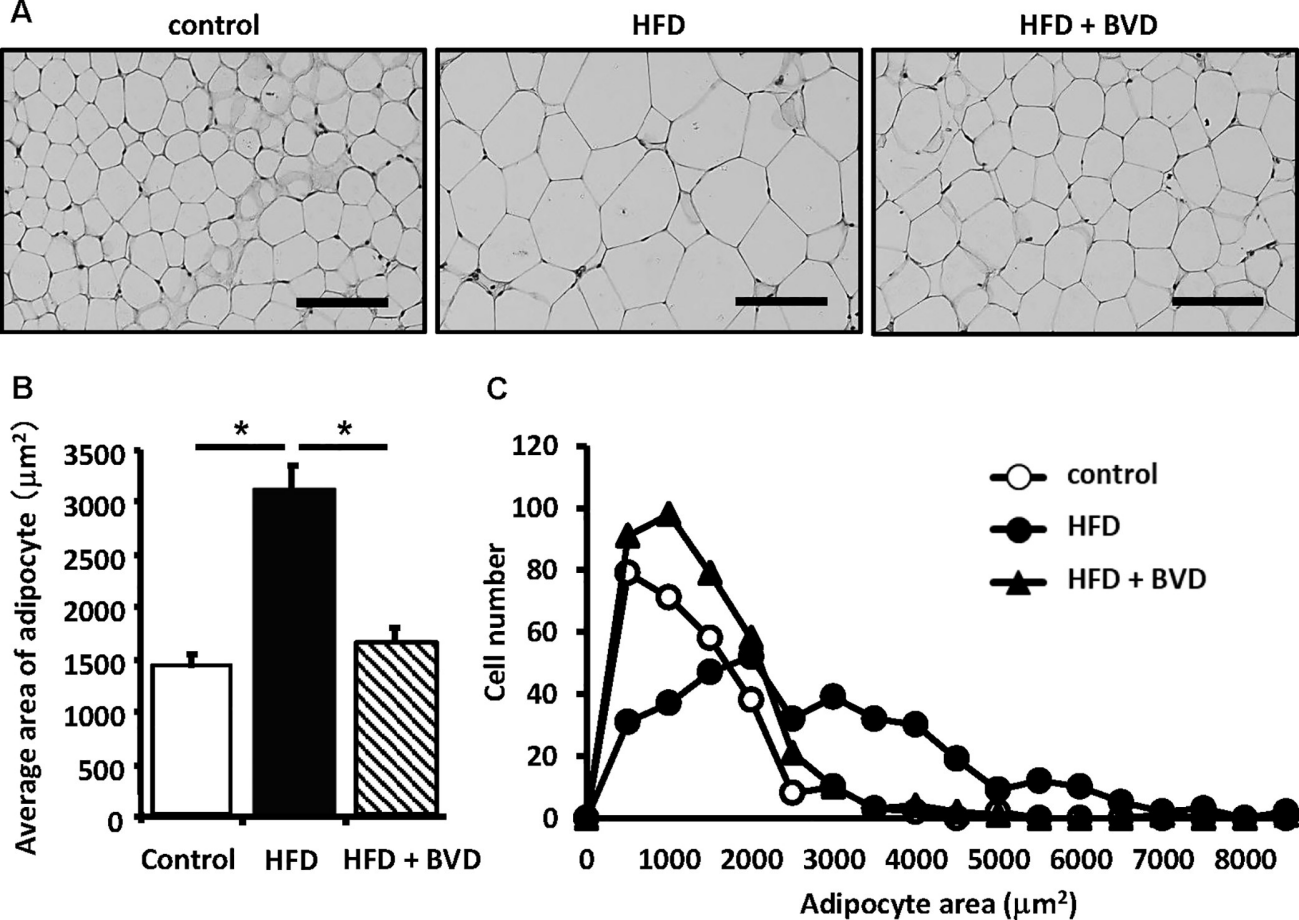
Effects of biliverdin (BVD) on adipocyte size. Representative photomicrographs (A), mean adipocyte area (B), and histogram of adipocyte area (C) derived from adipose tissue sections stained with haematoxylin and eosin. One hundred adipocytes per mouse were used for quantitative evaluation. Control mice (○), HFD mice (●), and HFD mice treated with BVD (▲). Results are expressed as means ± SEM (n = 3). Scale bar, 100 μm. Original magnification, ×200. *P < 0.01 (ANOVA).

**Fig 3:**
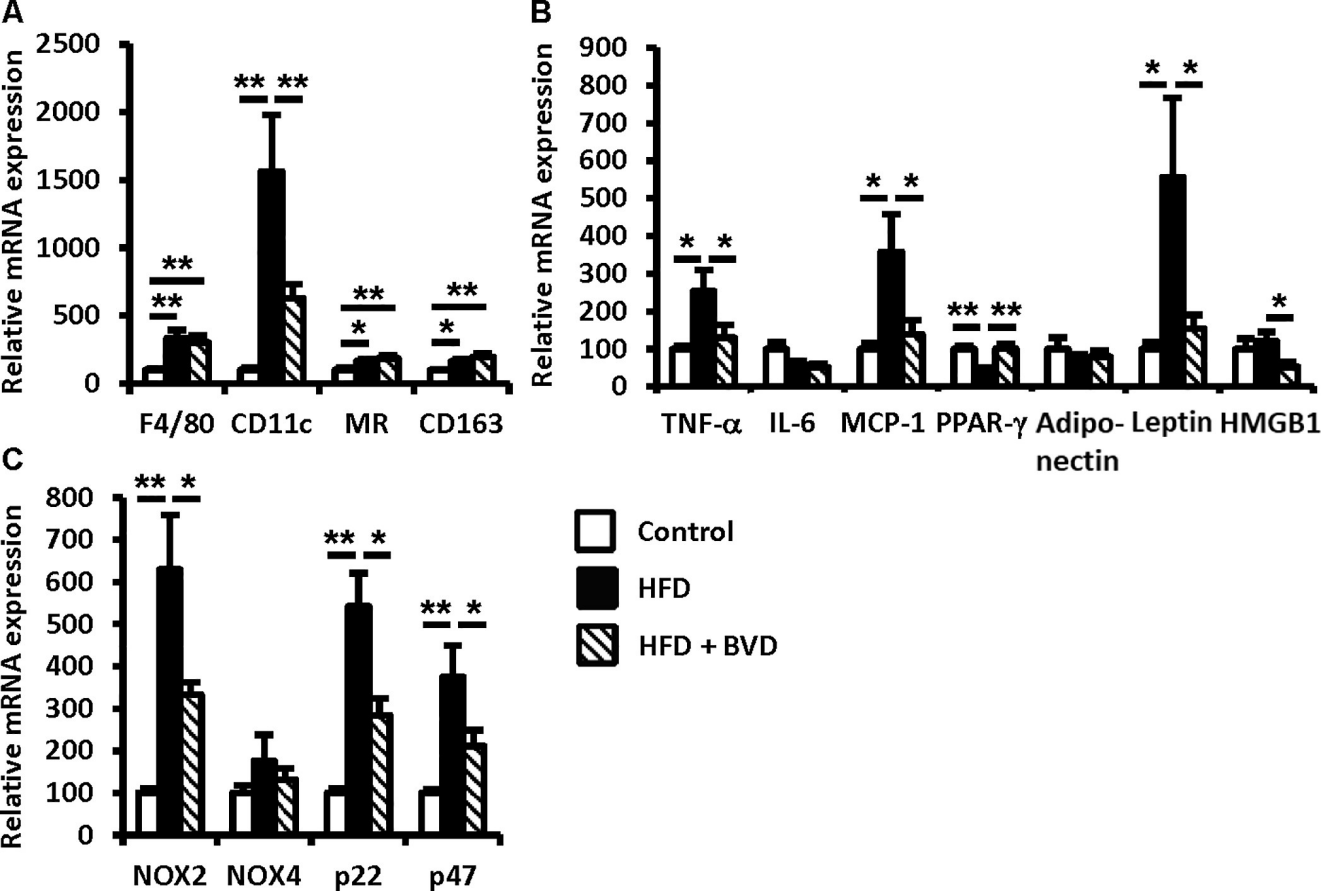
Effect of biliverdin (BVD) on gene expression. The expressions of macrophage markers (A), adipocytokines (B), master regulators of adipogenesis (B), and NAD(P)H oxidase components (C) were measured in white adipose tissue. Total RNA was extracted from the white adipose tissues of control mice (open bars), high-fat diet (HFD)-fed mice (closed bars) and HFD mice treated with biliverdin (BVD) (hatched bars). mRNA expression was measured using real-time RT-PCR and normalized to the expression of *β-actin*. Results are expressed as means ± SEM. (n = 8). *P < 0.05 and **P < 0.01 (ANOVA).

**Fig 4:**
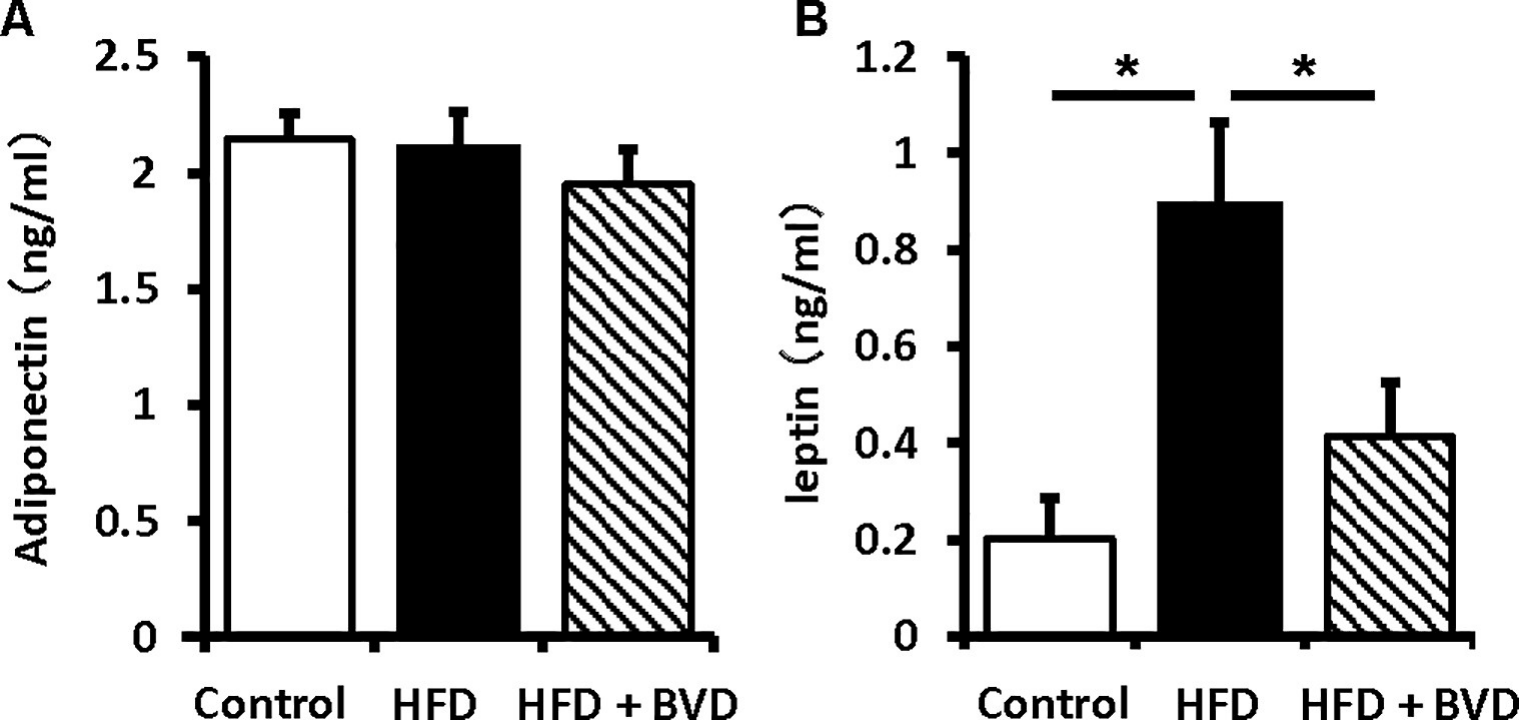
Serum concentrations of adiponectin and leptin. Control mice (open bars), high-fat diet (HFD)-fed mice (closed bars) and HFD mice treated with biliverdin (BVD) (hatched bars). Results are expressed as means ± SEM (n = 8). *P < 0.05 (ANOVA).

To determine the effect of biliverdin treatment on adipose tissue inflammation, we undertook several studies using WAT. The mean adipocyte size was higher in untreated HFD-fed mice than in CD-fed mice, but this difference was eliminated by biliverdin treatment (Fig 2). We next determined whether biliverdin treatment altered macrophage polarization. The mRNA expressions of *ADGRE1*, a pan-macrophage maker, and *Cd11c*, an M1 macrophage marker, were significantly higher in untreated HFD-fed mice than in CD-fed mice, but biliverdin-treated mice had a lower *Cd11c* expression (Fig 3A). In contrast, biliverdin treatment did not affect the mRNA expressions of mannose receptor (*MR*) and *Cd163*, M2 macrophage markers (Fig 3A). In addition, the mRNA expression of monocyte chemoattractant protein-1 (*Mcp-1*) was higher in untreated HFD-fed mice than in CD-fed mice, but this difference was abolished by biliverdin treatment. We next measured the mRNA expressions of *tumor necrosis factor-α* (*Tnf*a) and *Il6*, encoding cytokines that are considered to be important mediators of insulin resistance in obesity [20, 21]. As shown in Fig 3B, *Tnfa* mRNA expression was higher in untreated HFD-fed mice than in CD-fed mice, and this difference was abolished by biliverdin treatment; however, there were no significant differences in the mRNA expression of *Il6*. Next, we evaluated *high-mobility group box-1* (*HMGB1*), which is derived by adipose tissues and acts as an important proinflammatory mediator [22]. *HMGB1* mRNA expression was lower in HFD-fed mice treated with biliverdin than in HFD-fed mice. Finally, we measured the expression of genes encoding adipocytokines and their upstream transcriptional regulators. Interestingly, the mRNA expression of *peroxisome proliferator-activated receptor-γ* (*Ppparg*) was significantly lower in untreated HFD-fed mice than in CD-fed mice, and this difference was eliminated by biliverdin treatment (Fig 3B). Consistent with this finding, the mRNA expression and serum concentration of leptin were significantly higher in untreated HFD-fed mice than in CD-fed mice, and this difference was abolished by biliverdin treatment. However, there were no significant differences in the mRNA expression and serum concentration of adiponectin (Figs 3B and 4).

#### Effect of biliverdin treatment on the expression of NAD(P)H oxidase

Bilirubin is an endogenous antioxidant [13]. Therefore, we measured the expression of components of NAD(P)H oxidase (*P22phox*, *Gp91phox* (*Nox2*), and *P47phox*), a source of superoxide, and found that it was markedly higher in untreated HFD-fed mice compared with controls, and that this effect was ameliorated by biliverdin treatment. However, there were no significant differences in the mRNA expression level of *Nox4*, another NAD(P)H oxidase component (Fig 3C). These data suggest that biliverdin may inhibit oxidative stress in WAT by limiting the upregulation of NAD(P)H oxidase expression.

## Discussion

We showed that serum bilirubin concentration was inversely correlated with BMI, WC, TG, UA, Cre, and VFA in a cohort of patients with type 2 diabetes mellitus. The inverse relationship between serum bilirubin and VFA was also present after adjustment for other factors, implying that the association is independent. However, there was no association between SFA and serum bilirubin concentration. These results suggest that serum bilirubin concentration may affect body fat distribution in patients with diabetes mellitus. However, a cause-and-effect relationship cannot be ascribed on the basis of data collected during a cross-sectional study. Therefore, we performed an animal study to explore the mechanism underpinning these associations.

Because biliverdin is more water soluble than bilirubin, we treated mice orally with biliverdin in the current animal study. As biliverdin rapidly enters cells, it is converted to bilirubin by biliverdin reductase [23]. Therefore, increased intracellular bilirubin levels might have the beneficial effects of biliverdin treatment. Consistent with the results of the clinical study, we found that biliverdin treatment reduced adipocyte size and improved HFD-induced insulin resistance. In a previous study, we showed that biliverdin administration protected against diabetic nephropathy and pancreatic beta cell deterioration in diabetic mice via the inhibition of oxidative stress [15, 24], although serum bilirubin levels were not increased. These studies suggested that biliverdin administration inhibited oxidative stress via the increased intracellular bilirubin levels generated by biliverdin reductase. The current study indicated that biliverdin administration might have a beneficial effect on adipocyte size expansion, inflammation, and the dysregulation of adipocytokine expression in adipose tissues via these mechanisms; however, further studies are necessary.

Insulin resistance is associated with chronic inflammation in adipose tissue, which involves a switch in adipose tissue macrophage (ATM) polarization [25–27]. Macrophages can be characterized into a proinflammatory (M1 macrophages) or anti-inflammatory phenotype (M2 macrophages). M1 macrophages are host-defense cells that kill pathogens and secrete proinflammatory cytokines such as TNF-α and IL-6 [28], both of which contribute to insulin resistance [20, 21]. In contrast, M2 macrophages dampen these proinflammatory and adaptive T-helper 1 responses by secreting anti-inflammatory cytokines (IL-10, transforming growth factor-β (TGF-β) and IL-1 receptor antagonist) [28]. In the current study, biliverdin treatment reduced the expressions of M1 markers, *Tnfa* and *HMGB1* in adipose tissues induced by HFD-feeding. These data imply that bilirubin may improve HFD-induced insulin resistance by reducing chronic inflammation in adipose tissue.

In the current study, we showed that the higher expression of *Mcp-1* and the lower expression of *Ppparg* in HFD-fed mice was ameliorated by biliverdin treatment. Consistent with our results, Liu et al. reported that bilirubin administration increased *Ppparg*, which reduced the size of adipocytes in epididymal fat and hepatic lipid accumulation in DIO mice [29]. The mRNA expression and serum concentration of leptin were at their optimal levels for insulin sensitivity. These data suggest that bilirubin may influence adipocyte size and ATM polarization in HFD-fed mice by altering the expressions of regulators of adipogenesis and adipocytokines. A previous report proposed that greater oxidative stress induced by NADPH oxidase activation led to the dysregulated production of adipocytokines (fat-derived hormones) and regulators of adipogenesis including adiponectin, IL-6, PPAR-γ, and MCP-1 [30]. In the current study, we showed that biliverdin treatment decreased the high mRNA expressions of components of NAD(P)H oxidase, a major source of superoxide, in HFD-fed mice. Therefore, biliverdin treatment may improve HFD-induced insulin resistance by promoting a favorable adipocytokine profile *via* the inhibition of oxidative stress. It is also possible that biliverdin administration might alleviate insulin resistance through activated PPAR-α signaling, because recent studies reported that bilirubin activated PPAR-α [31–34]. However, more detailed assessments of the molecular mechanisms involved should be made in future studies. One limitation of our human study was the median BMI was 25.5 kg/m^2^ (IQR, 22.1–28.9 kg/m^2^) in the enrolled type 2 diabetes mellitus patients, although we used a HFD-induced obese mice model in animal study. Therefore, we should also examine an obese patient group (BMI > 30 kg/m^2^) in a future clinical study.

In conclusion, this study has shown that serum bilirubin concentration is inversely correlated with VFA in patients with diabetes mellitus, and that biliverdin administration alleviates insulin resistance, potentially by ameliorating adipose tissue inflammation, adipocyte expansion, and the dysregulation of adipocytokines in HFD-fed mice. Thus, our findings suggest that bilirubin may ameliorate visceral obesity and insulin resistance, which identifies it as a potential target for novel therapies to protect against insulin resistance in patients with visceral obesity and diabetes.

## Supporting information

S1 Table Conditions and specific primers for real-time PCR methods. (DOC)

S2 Table Conditions and specific primers for real-time PCR methods. (DOC)

S3 Table Conditions and specific primers for real-time PCR methods. (DOC)

S4 Table Correlations between body fat distribution and other variables. (DOC)

S1 Fig Experimental protocol and body weight. Experimental protocol (A) and changes in body mass (B) between 8 and 18 weeks of age in control mice (○), high-fat diet (HFD)-fed mice (●) and HFD-fed mice treated with biliverdin (BVD) (▲). Results are expressed as the mean ± SEM (n = 8); *P < 0.05 *vs* control mice (ANOVA).
(TIF)

S1 File STROBE statement. Checklist of items that should be included in reports of observational studies.
(DOCX)
