# Supplementary material for: Bilirubin reduces visceral obesity and insulin resistance by suppression of inflammatory cytokines
Source: PLoS One. 2019 Oct 2;14(10):e0223302. doi: 10.1371/journal.pone.0223302 (PMC6774504; doi:10.1371/journal.pone.0223302)
Supplement: S1 Table — (DOC) [file pone.0223302.s001.doc]

| Target mRNA | *ADGRE1* | *CD-11c* | *MR* | *CD163* | *TNF-*α | | *IL-6* | *MCP-1* |
| --- | --- | --- | --- | --- | --- | --- | --- | --- |
| Preincubation | 95ºC, 30 s | | | | | | | |
| PCR | 92**º**C, 1 s | | | | | 40 cycles | | |
|  | 62ºC, 10 s | | | | |  |  |  |
|  | 72**º**C, 15 s | | | | |  |  |  |
| Sense primer | 5’- CCT GGA  CGA ATC CTG  TGA AG-3’ | 5’- GAA GAG  CCA CTT CCC  AAC TG-3’ | 5’-CCA CAG  CAT TGA GGA  GTT TG-3’ | 5’-CCT GGA  TCA TCT GTG  ACA ACA-3’ | 5’-TCT TCT  CAT TCC TGC  TTG TGG-3’ | | 5’-GAT GGA  TGC TAC CAA  ACT GGA-3’ | 5’-CAT CCA  CGT GTT GGC  TCA-3’ |
| Antisense primer | 5’- GGT GGG  ACC ACA GAG  AGT TG-3’ | 5’- TCA GGA  ACA CGA TGT  CTT GG-3’ | 5’-ACA GCT  CAT CAT TTG  GCT CA-3’ | 5’-TCC ACA  CGT CCA GAA  CAG TC-3’ | 5’-GGT CTG  GGC CAT AGA  ACT GA-3’ | | 5’-CCA GGT  AGC TAT GGT  ACT CCA GAA-3’ | 5’-GAT CAT  CTT GCT GGT  GAA TGA GT-3’ |

**Supplemental Table 1. Conditions and specific primers for real-time PCR methods**

PCR, polymerase chain reaction; mRNA, messenger RNA; MR, mannose receptor; TNF-α, tumor necrosis factor-α; MCP-1, monocyte chemoattractant protein-1.
