# Supplementary material for: Bilirubin reduces visceral obesity and insulin resistance by suppression of inflammatory cytokines
Source: PLoS One. 2019 Oct 2;14(10):e0223302. doi: 10.1371/journal.pone.0223302 (PMC6774504; doi:10.1371/journal.pone.0223302)
Supplement: S2 Table — (DOC) [file pone.0223302.s002.doc]

**Supplemental Table 2. Conditions and specific primers for real-time PCR methods**

| Target mRNA | *PPAR-γ* | *Adiponectin* | *Leptin* | *NOX2* | *NOX4* | | *p22* | *p47* |
| --- | --- | --- | --- | --- | --- | --- | --- | --- |
| Preincubation | 95ºC, 30 s | | | | | | | |
| PCR | 92**º**C, 1 s | | | | | 40 cycles | | |
|  | 62ºC, 10 s | | | | |  |  |  |
|  | 72**º**C, 15 s | | | | |  |  |  |
| Sense primer | 5’-CTG TGA  GAC CAA CAG  CCT GA-3’ | 5’-GTT GCA  AGC TCT CCT  GTT CC-3’ | 5’-TGA CAC  CAA AAC CCT  CAT CA-3’ | 5’-ACT GCG  GAG AGT TTG  GAA GA-3’ | 5’-ATT TGG  ATA GGC TCC  AGG CAA AC-3’ | | 5’-TGG CTA  CTG CTG GAC  GTT TCA C--3’ | 5’-TGC CTC  CAT AAG ATC  TTG GTG ATT-3’ |
| Antisense primer | 5’-AAT GCG  AGT GGT CTT  CCA TC-3’ | 5’-TCT CCA  GGA GTG CCA  TCT CT-3’ | 5’-TCA TTG  GCT ATC TGC  AGC AC-3’ | 5’-GGT GAT  GAC CAC CTT  TTG CT-3’ | 5’-CAC ATG  GGT ATA AGC  TTT GTG AGC A-3’ | | 5’-CTC CAG  CAG ACA GAT  GAG CAC AC-3’ | 5’-CTT GCT  CAG CCT GCC  TTC TTA TAG-3’ |

PPAR-γ, peroxisome proliferator-activated receptor-γ.
