# Supplementary material for: Bilirubin reduces visceral obesity and insulin resistance by suppression of inflammatory cytokines
Source: PLoS One. 2019 Oct 2;14(10):e0223302. doi: 10.1371/journal.pone.0223302 (PMC6774504; doi:10.1371/journal.pone.0223302)
Supplement: S3 Table — (DOC) [file pone.0223302.s003.doc]

**Supplemental Table 3. Conditions and specific primers for real-time PCR methods**

| Target mRNA | *HMGB1* |  |
| --- | --- | --- |
| Preincubation | 95ºC, 30 s | |
| PCR | 92**º**C, 1 s | 40 cycles |
|  | 62ºC, 10 s |  |
|  | 72**º**C, 15 s |  |
| Sense primer | 5’-GGA GTG  GCT TTT GTC  CCT CAT-3’ |  |
| Antisense primer | 5’-TGC CTC  TCG GCT TTT  TAG GA-3’ |  |

HMGB1, high-mobility group box-1
