# Supplementary material for: Bilirubin reduces visceral obesity and insulin resistance by suppression of inflammatory cytokines
Source: PLoS One. 2019 Oct 2;14(10):e0223302. doi: 10.1371/journal.pone.0223302 (PMC6774504; doi:10.1371/journal.pone.0223302)
Supplement: S4 Table — (DOC) [file pone.0223302.s004.doc]

**Supplemental Table 4. Correlations between body fat distribution and other variables**

|  | VFA | | | |  | SFA | | | |
| --- | --- | --- | --- | --- | --- | --- | --- | --- | --- |
|  | Univariate | | Multivariate | |  | Univariate | | Multivariate | |
| Variables | ρ | p value | β | p value |  | ρ | p value | β | p value |
| Age | -0.108 | 0.154 | 0.173 | 0.021 |  | -0.326 | 0.001 | -0.018 | 0.672 |
| Sex, female | -0.219 | 0.004 | -0.132 | 0.013 |  | -0.002 | 0.982 | 0.058 | 0.159 |
| Body mass index | 0.790 | 0.001 | 0.587 | 0.001 |  | 0.909 | 0.001 | 0.892 | 0.001 |
| SBP | 0.252 | 0.001 | -0.020 | 0.750 |  | 0.220 | 0.003 | -0.026 | 0.595 |
| DBP | 0.363 | 0.001 | 0.115 | 0.078 |  | 0.371 | 0.001 | 0.030 | 0.554 |
| Fasting plasma glucose | 0.102 | 0.177 | 0.051 | 0.357 |  | 0.090 | 0.235 | 0.046 | 0.285 |
| HbA1c | -0.079 | 0.296 | -0.143 | 0.013 |  | -0.002 | 0.980 | 0.007 | 0.866 |
| Total cholesterol | 0.092 | 0.223 | 0.045 | 0.468 |  | 0.186 | 0.013 | 0.021 | 0.657 |
| HDL-C | -0.327 | 0.001 | -0.251 | 0.001 |  | -0.140 | 0.063 | 0.028 | 0.505 |
| TG | 0.509 | 0.001 | 0.030 | 0.625 |  | 0.316 | 0.001 | -0.037 | 0.444 |
| UA | 0.319 | 0.001 | 0.080 | 0.148 |  | 0.157 | 0.038 | -0.009 | 0.836 |
| Bilirubin | -0.191 | 0.011 | -0.100 | 0.045 |  | -0.130 | 0.852 | -0.026 | 0.502 |
| Cre | 0.194 | 0.010 | -0.116 | 0.029 |  | -0.005 | 0.947 | -0.067 | 0.101 |

VFA, visceral fat area; SFA, subcutaneous fat area; SBP, systolic blood pressure; DBP, diastolic blood pressure; HbA1c, hemoglobin A1c; HDL-C, high-density lipoprotein cholesterol; TG, triglycerides; UA, uric acid; Cre, creatinine.
