# Supplementary figures and images for: Bilirubin reduces visceral obesity and insulin resistance by suppression of inflammatory cytokines
Source: PLoS One. 2019 Oct 2;14(10):e0223302. doi: 10.1371/journal.pone.0223302 (PMC6774504; doi:10.1371/journal.pone.0223302)

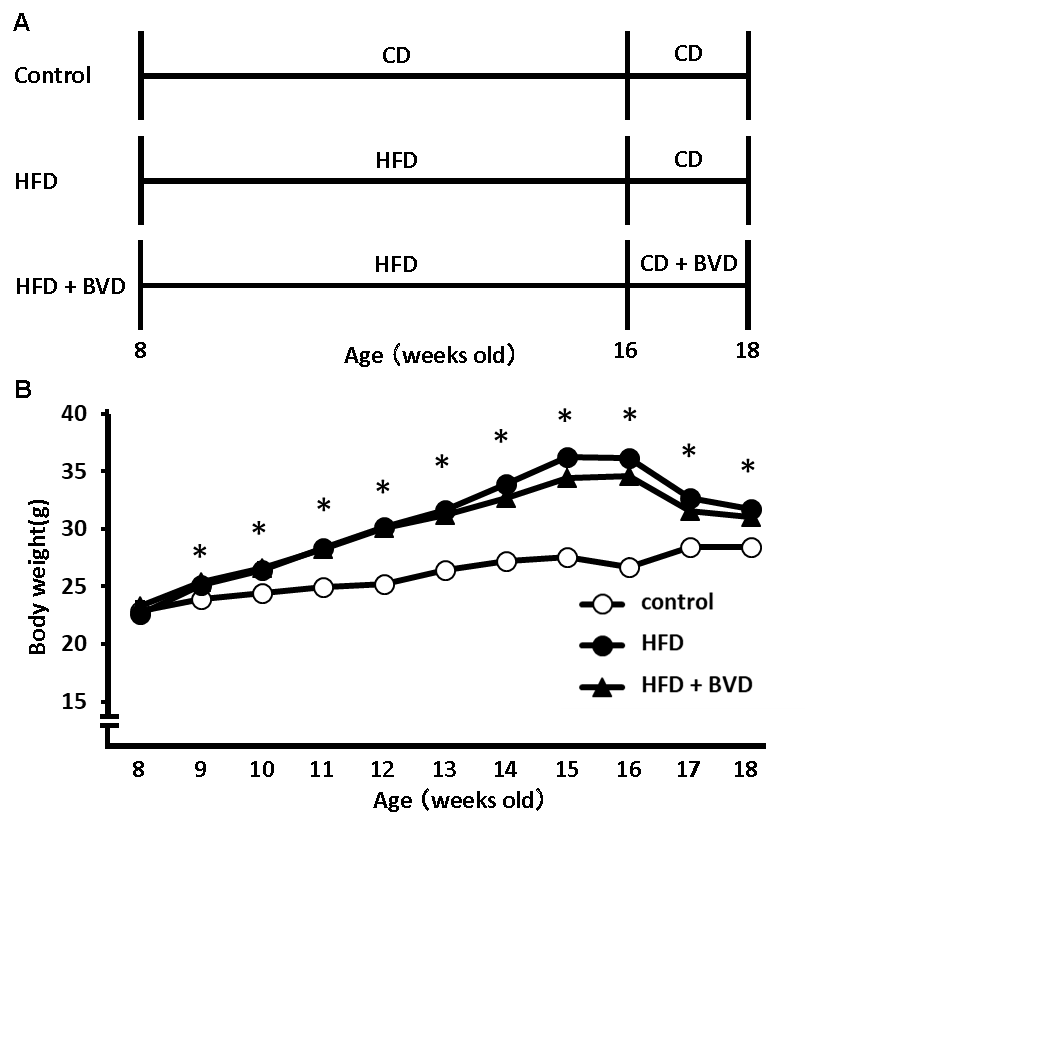

Supplement: S1 Fig — Experimental protocol (A) and changes in body mass (B) between 8 and 18 weeks of age in control mice (○), high-fat diet (HFD)-fed mice (●) and HFD-fed mice treated with biliverdin (BVD) (▲). Results are expressed as the mean ± SEM (n = 8); *P < 0.05 vs control mice (ANOVA). (TIF) [file pone.0223302.s005.tif]
